# The Concomitant Use of Atorvastatin and Amlodipine Leading to Rhabdomyolysis

**DOI:** 10.7759/cureus.2020

**Published:** 2018-01-03

**Authors:** Salman Khan, Imran Khan, Matthew Novak, Asish Regmi, William Difilippo

**Affiliations:** 1 Internal Medicine, Guthrie Clinic/Robert Packer Hospital; 2 Department of Cardiology, Hofstra North Shore-LIJ School of Medicine, North Shore University Hospital; 3 Nephrology, Guthrie Clinic/Robert Packer Hospital

**Keywords:** statin, calcium channel blockers, rhabdomyolysis, cholesterol, kidney

## Abstract

A 65-year-old man, with a history of hypertension and hyperlipidemia, presented with intractable lower back pain, shortness of breath, and decreasing urine output at the emergency room and was admitted after he was found to have elevated creatinine kinase levels of greater than 160,000 U/L. We discontinued all his home medications, which included atorvastatin and amlodipine. We trended his creatine phosphokinase (CPK) level daily and noticed it decreasing significantly off these meds. We hydrated him with normal saline and monitored his kidney functions. By the time he was ready for discharge, his CPK levels were back to normal. This case report summarizes the drug-drug interactions of atorvastatin and amlodipine.

## Introduction

The coadministration of amlodipine is generally safe when administered with 3-hydroxy-3-methyl-glutaryl-coenzyme A (HMG COA) reductase inhibitors. Our patient has been on both medications together for several years, with one previous episode of elevated creatine kinase. Despite that, his home medications were not investigated and continued until this issue recurred, earning him a trip to the emergency room. With this in mind, there is a possibility that he had the HMG COA antibody, a new novel approach to patients with rhabdomyolysis post statin use. Our case showed evidence of combining a statin and calcium channel blocker, causing rhabdomyolysis in a patient with no provocation. The patient was on statins for six years, combined with a calcium channel blocker for at least four years.

## Case presentation

A 65-year-old man with a history of chronic kidney disease stage three, chronic obstructive pulmonary disease, hypertension, and benign prostatic hyperplasia presented to the emergency room with lower back pain, red-colored urine, and decreased urination for the past week. He said there had been no changes in his activity or his medication administration recently. A computed tomography (CT) of the abdomen was performed, which revealed an inflamed psoas muscle. The patient also had blood work conducted, which showed a creatine kinase level greater than 160,000 U/L. The patient was admitted and his home medications, which included atorvastatin and amlodipine, were discontinued. The following seven days, we trended his creatine kinase daily, which showed significant downward trends, starting from 160,000 U/L early in the admission down to 5000 U/L before discharge. After going through his medical history for the last few years, he once had an elevated creatine kinase previously that was misdiagnosed as a viral infection. Interestingly, he was started on amlodipine while he was on atorvastatin. We monitored him for seven days with serial creatine kinase and renal function panels daily and noted his creatine kinase trending down. Once he was close to his normal creatine kinase level and asymptomatic, we discharged him with no statin therapy and had him follow up in two weeks with nephrology.

## Discussion

Statins are commonly prescribed lipid-lowering medications that reduced the risk of cardiovascular-related issues. However, they may have mild toxic effects that can range from muscle aches, such as myalgias to rhabdomyolysis, which is muscle inflammation or breakdown. Patients on statins are at an increased risk for rhabdomyolysis, particularly those with a slow metabolism that may be related to gene variation with the hepatic statin transporter [1]. 

The combination of dihydropyridine calcium channel blockers with statins is commonly used by clinicians for patients with high cholesterol and hypertension. To reduce this risk of myopathy, the Food and Drug Administration (FDA) set a new dose limitation for simvastatin for patients who were also taking amlodipine. However, there was no dose limitation for atorvastatin for patients who are also taking amlodipine. Atorvastatin is commonly prescribed for patients with hypercholesterolemia that are at an increased risk of adverse cardiovascular disease. Atorvastatin is widely used for hypercholesterolemia and cardiovascular disease. These medications metabolize cytochrome p450 3A4. The Food and Drug Administration has enforced that people taking simvastatin with amlodipine should take a maximum dose of no more than 20 mg because of the risk of myopathy. No dose limitation has been suggested for patients taking atorvastatin while they are also taking amlodipine [2]. However, the risk of statin-induced myopathy due to cytochrome p450 inhibitors appears considerably greater when taking lovastatin and simvastatin compared to atorvastatin. Atorvastatin does not undergo an extensive pre-systemic metabolism, such as lovastatin or simvastatin. Potent cytochrome p450 3A4 tends to produce two- to four-fold increases in atorvastatin C max concentrations [2]. Combining statins with cytochrome p450 inhibitors undergoes extensive pre-systemic metabolisms in the gut wall and liver [3].

Rhabdomyolysis is characterized by severe acute muscle injury, resulting in muscle pain, weakness, or inflammation with muscle fiber contents spilling into the bloodstream, which can darken the urine, resulting in myoglobinuria. Serum creatinine kinase and urine myoglobin levels are usually elevated. Clinical exams, history taking, and lab studies are all helpful in the diagnosis of rhabdomyolysis but muscle biopsy and genetic testing are also useful tools, and they can help differentiate between acquired and inherited rhabdomyolysis. Acquired causes can include substance abuse medication or toxic exposure as well as electrolyte abnormalities, endocrine disturbance, or autoimmune myopathies. The diagnosis of rhabdomyolysis is based primarily on seeing myoglobin in the urine or elevated serum creatinine kinase. The plasma myoglobin increases rapidly and then is cleared out by the kidney. Normal levels can be re-established or retained after 24 hours. Serum creatinine levels can rise within two to 12 hours after injury and continue to rise up to five days after injury and then decline over the next two weeks. Not all patients will present at the time of the initial injury, which questions when the injury took place vs. when they arrived in the emergency room [4].

Itraconazole is another potent inhibitor of cytochrome p450 and increases the toxicity of HMG COA reductase inhibitors by increasing their serum concentrations. Itraconazole can increase atorvastatin plasma levels, suggesting that an itraconazole and atorvastatin combined therapy should be very carefully monitored. Itraconazole combined with cerivastatin may be preferable [5].

## Conclusions

This case illustrates that when high doses of atorvastatin are co-administered with amlodipine as a secondary prevention for cardiovascular risk, the combination may pose a risk for serious myotoxicity, such as rhabdomyolysis. In this article, atorvastatin poses a risk for rhabdomyolysis alone and adding amlodipine may further increase the risk of statin-associated rhabdomyolysis. Further awareness of statin-associated risks should always be raised in susceptible patients. Close monitoring and dose alteration are recommended when these medications are co-administered.
